# BRANEart: Identify Stability Strength and Weakness Regions in Membrane Proteins

**DOI:** 10.3389/fbinf.2021.742843

**Published:** 2021-12-02

**Authors:** Sankar Basu, Simon S. Assaf, Fabian Teheux, Marianne Rooman, Fabrizio Pucci

**Affiliations:** ^1^ Computational Biology and Bioinformatics, Université Libre de Bruxelles, Brussels, Belgium; ^2^ Department of Microbiology, Austosh College, Under University of Calcutta, Kolkata, India; ^3^ Interuniversity Institute of Bioinformatics in Brussels, Brussels, Belgium

**Keywords:** computational prediction, membrane protein structure and stability, folding free energy, frustration, statistical potentials, Leucine Transporters, Human Phospholamban

## Abstract

Understanding the role of stability strengths and weaknesses in proteins is a key objective for rationalizing their dynamical and functional properties such as conformational changes, catalytic activity, and protein-protein and protein-ligand interactions. We present BRANEart, a new, fast and accurate method to evaluate the per-residue contributions to the overall stability of membrane proteins. It is based on an extended set of recently introduced statistical potentials derived from membrane protein structures, which better describe the stability properties of this class of proteins than standard potentials derived from globular proteins. We defined a per-residue membrane propensity index from combinations of these potentials, which can be used to identify residues which strongly contribute to the stability of the transmembrane region or which would, on the contrary, be more stable in extramembrane regions, or *vice versa*. Large-scale application to membrane and globular proteins sets and application to tests cases show excellent agreement with experimental data. BRANEart thus appears as a useful instrument to analyze in detail the overall stability properties of a target membrane protein, to position it relative to the lipid bilayer, and to rationally modify its biophysical characteristics and function. BRANEart can be freely accessed from http://babylone.3bio.ulb.ac.be/BRANEart.

## 1 Introduction

The broad family of integral membrane proteins provides indispensable components of living cells. Being embedded in biological lipid membranes, these proteins include important scaffolds and functional sites, which bind targeted molecules floating around in the cytosol or in the extracellular medium. They therefore serve as attractive drug targets (Engel and Gaub, 2008; Cournia et al., 2015).

Stability and physico-chemical characteristics greatly vary between transmembrane (TM) and extramembrane (EM) regions of an integral membrane protein due to the difference in their surrounding chemical environment. EM domains in the cytosolic or extracellular medium resemble globular proteins as their surfaces are exposed to water, while residues embedded within the membrane are exposed to lipids and are thus characterized by an elevated hydrophobicity (Leman et al., 2018; Mbaye et al., 2019). Because of this mixed environment, the study of folding and stability of membrane proteins is very challenging and only few tools have been dedicated to investigate them (Lomize et al., 2012; Alford et al., 2015; Postic et al., 2015; Lu et al., 2018). Another reason why membrane proteins are less studied than globular proteins is their lower number of experimental three-dimensional (3D) structures (Shimizu et al., 2018).

Just as for globular proteins, the native structure of a membrane protein corresponds to the global minimum of the free energy landscape in physiological conditions. In general, however, some protein residues or regions, taken individually, are not in their global free energy minimum and correspond to what we call stability weaknesses (Kwasigroch and Rooman, 2006; De Laet et al., 2016; Hou et al., 2021). Indeed, not all residues can simultaneously adopt their lowest free energy conformations, because of the polypeptide chain which constraints the relative motions of the residues. Native conformations can be viewed as the best compromise between conflicting interactions. We define strong and weak residues as residues that are, or are not, optimized for stability properties, respectively. For example, stability weaknesses occur when a contact between two residues in the native structure does not have a favorable free energy contribution and can thus easily break, change conformation, and/or remain flexible. They often play a key role in protein function, interactions, and conformational changes. The concept of stability weaknesses is close to the notion of frustration except that the latter also includes kinetic constraints on fast folding (Ferreiro et al., 2014).

To further advance these issues, we implemented and expanded a series of new statistical mean-force potentials designed to specifically describe the stability properties of membrane proteins, which were first introduced in Mbaye et al. (2019). Based on these potentials, we defined here a per-residue membrane propensity index which predicts whether residues situated in the membrane have a stabilizing contribution or would prefer to be in EM regions, and similarly for residues outside the membrane. This index thus identifies strong and weak residues in EM and TM regions, and can also be used to predict how a protein is, or is not, inserted in the membrane.

It is to be noted that we are studying here relative, EM/TM, strengths and weaknesses, defined from the difference in folding free energy according to whether a residue is in one or the other region. They are different from the strengths and weaknesses defined as residues of which the folding free energy contribution is either highly optimal or not optimal at all in a given environment, as for example computed by the SWOTein web server for globular proteins (Hou et al., 2021). These quantities are related but nevertheless different and give complementary information.

We made our computational tool freely available in the form of a web server called BRANEart, designed to help the scientific community to explore stability strengths and weaknesses in membrane proteins, which is a key element in the study of their stability and function.

## 2 Materials and Methods

### 2.1 Protein Structure Data Sets

The membrane protein data set $D_{\text{mem}}$ comes directly from a recent study (Mbaye et al., 2019). It consists of 163 X-ray structures of integral membrane proteins that have been collected from the Protein Data Bank (PDB) (Berman et al., 2000). The selected structures have a resolution of at most 2.5 Å  and a pairwise sequence identity of at most 30%, computed using the PISCES protein sequence culling server (Wang and Dunbrack Jr, 2003).

The set $D_{\text{mem}}$ contains 107 *α*-helical and 52 *β*-barrel polytopic membrane, and 4 *α*-helical monotopic proteins that do not span the lipid bilayer completely. Each of these proteins was annotated using OPM (Orientations of Proteins in Membranes) (Lomize et al., 2012), a curated web resource that positions the biological lipid bilayer on experimentally resolved structures of integral membrane proteins and membrane-bound peptides. Using these annotations, the TM and EM portions of each protein were further segregated in two subsets $D_{\text{mem}}^{\text{TM}}$ and $D_{\text{mem}}^{\text{EM}}$ .

All selected protein chains in $D_{\text{mem}}$ were considered in the context of their biological assembly (or biounit), which corresponds to their functional quaternary conformation. This ensures a more realistic representation of the surrounding protein environment experienced by the protein. The biological units were taken to be those defined by the authors of the X-ray structures or, in absence of author annotations, as those predicted by the Protein Interfaces, Surfaces and Assemblies (PISA) tool (Krissinel and Henrick, 2007).

Finally, we also set up a second data set $D_{\text{glob}}$ of 4,860 monomeric globular protein structures from the PDB, to be used as an independent set for validating our method. The proteins from this data set have a monomeric biological unit, a good quality X-ray structure with a maximum resolution of 2.5 Å  and a pairwise sequence identity of at most 25%.

The list of all proteins belonging to the data sets $D_{\text{mem}}$ and $D_{\text{glob}}$ are given in the GitHub repository https://github.com/3BioCompBio/BRANEart.

### 2.2 Statistical Potentials

Statistical potentials are coarse-grained mean-force energy functions derived from frequencies of associations of sequence and structure motifs in a data set of known protein structures. These frequencies are transformed into free energies using the inverse Boltzmann law (Sippl, 1990; Kocher et al., 1994; Dehouck et al., 2006). These potentials depend on the characteristics of the data set from which they are derived. For example, temperature-dependent statistical potentials are obtained from protein data sets of different melting temperatures (Folch et al., 2010; Pucci et al., 2014), and solubility-dependent potentials from proteins of different solubility (Hou et al., 2018).

Here we derived a series of membrane protein potentials from the sets $D_{\text{mem}}^{\text{TM}}$ and $D_{\text{mem}}^{\text{EM}}$ , extending the work of Mbaye et al. (2019). More precisely, we considered the following first and second order statistical potential terms (Dehouck et al., 2006):

$$\Delta W_{xy}=-k_{B}T\ln\frac{F\left(x,y\right)}{F\left(x\right)\;F\left(y\right)}$$ (1)

$$\Delta W_{xyz}=-k_{B}T\ln\frac{F\left(x,y,z\right)\;F\left(x\right)\;F\left(y\right)\;F\left(z\right)\;}{F\left(x,y\right)\;F\left(x,z\right)\;F\left(y,z\right)\;}$$ (2)

where *k*_*B*_ is the Boltzmann constant, *T*, the absolute temperature conventionally taken to be room temperature, and *F*, the relative frequencies computed from a given data set of protein structures. The variables *x*, *y*, *z* stand for any of the four elementary structure or sequence descriptors *s*, *d*, *t* and *a*: *s* is an amino acid type, *d*, the spatial distance between the average side chain geometric centers of two residues separated by at least one residue along the polypeptide chain (Kocher et al., 1994), *t*, a (*ϕ*, *ψ*, *ω*) backbone torsion angle domain (Rooman et al., 1991), and *a*, a solvent accessibility bin where the solvent accessibility is defined as the ratio (in %) between the solvent accessible surface area (ASA) of a residue in the structure and in an extended Gly-X-Gly conformation (Kocher et al., 1994).

We constructed two versions of each of the potentials defined by Eqs. 1, 2. In the first, all frequencies *F* were computed from the structure set $D_{\text{mem}}^{\text{TM}}$ , *i.e.,* considering only protein regions embedded in the lipid membrane. In the second, all frequencies *F* were computed from $D_{\text{mem}}^{\text{EM}}$ , thus considering only extramembrane protein regions. The potentials extracted from TM regions, $\Delta W_{\chi }^{\text{TM}}$ with *χ* = *xy* or *xyz*, describe the stability properties of membrane proteins inside the lipid membrane, while the potentials extracted from EM regions, $\Delta W_{\chi }^{\text{EM}}$ , describe the stability properties outside the lipid bilayer. Note that the only membrane protein potentials that we constructed and analyzed earlier are the inter-residue distance potentials $\Delta W_{sd}^{\mu }$ and $\Delta W_{sds}^{\mu }$ (Mbaye et al., 2019), where *μ* is either EM or TM.

The full list of 19 membrane statistical potentials derived here and their characteristics are given in Supplementary Material S1.

### 2.3 Per-Residue Folding Free Energies

The statistical potentials $\Delta W_{\chi }^{\mu }$ defined above are used to compute the contribution of each residue *i* in a given protein to its overall folding free energy $\Delta G_{\chi }^{i,\mu }$ . This is done by allocating an equal amount of energy to each of the interacting residues that carry the structural descriptors *a*, *t* and *d* included in *χ*. More precisely, we applied the following rules according to the structural descriptors used (De Laet et al., 2016; Hou et al., 2021):

• If the structure descriptors involved in *χ* are localized on a single residue *i* (which is the case when e.g., *χ* = *st*, *sa*, *sst*, *ssa*), the total contribution is assigned to that residue. For example, the per-residue folding free energy contribution $\Delta G_{st}^{i,\mu }$ can be written as
$$\Delta G_{st}^{i,\mu }=\overset{L}{\underset{j=1}{\sum }}\Delta W_{s_{j}t_{i}}^{\mu }\;$$ (3)
  where *s*_*j*_ denotes the amino acid type at position *j* and *t*_*i*_, the backbone torsion angle domain at position *i*; *L* is the sequence length. Note that the torsion and solvent accessibility descriptors *t* and *a* are required to be in a sequence windows of maximum 17 residues centered on the sequence descriptor *s*, thus |*i* − *j*| ≤ 8 (Supplementary Material S1). Outside this window, $\Delta W_{s_{j}t_{i}}^{\mu }=0$ .
• If the structure descriptor is localized on two residues *i* and *j* (e.g., *χ* = *sd*, *tt*, *aa*, *sds*, *saa*, *stt*), half of the energy contribution is assigned to each of the two residues. For example, the per-residue folding free energy $\Delta G_{sds}^{i,\mu }$ is:
$$\Delta G_{sds}^{i,\mu }=\frac{1}{2}\overset{L}{\underset{j=1}{\sum }}\Delta W_{s_{i}d_{ij}s_{j}}^{\mu }$$ (4)
  where *s*_*i*_ and *s*_*j*_ denote the amino acid types at positions *i* and *j*, respectively, and *d*_*ij*_ is the distance between residues *i* and *j* (Supplementary Figure S1A). Similarly, we have for the per-residue folding free energy $\Delta G_{saa}^{i,\mu }$ :
$$\Delta G_{saa}^{i,\mu }=\frac{1}{2}\overset{L}{\underset{j,k=1}{\sum }}\Delta W_{s_{k}a_{j}a_{i}}^{\mu }$$ (5)
  where *a*_*i*_ and *a*_*j*_ denote the solvent accessibility bin of residue *i* and *j*, respectively, and *s*_*k*_ is the amino acid type of residue *k*. Both descriptors *a*_*i*_ and *a*_*j*_ are required to be in a sequence window of maximum 17 residues centered on the sequence descriptor *s*_*k*_, thus |*i* − *k*| ≤ 8 and |*j* − *k*| ≤ 8 (Supplementary Material S1).
• For the potential *χ* = *ss* which does not contain any structure descriptor, an equal amount was allocated to each of the two residues carrying a sequence descriptor *s* (Supplementary Figure S1B):

$$\Delta G_{ss}^{i,\mu }=\frac{1}{2}\overset{L}{\underset{j=1}{\sum }}\Delta W_{s_{j}s_{i}}^{\mu }$$ (6)

For further details, we refer the reader to Supplementary Material S1, S2 and to previous studies (De Laet et al., 2016; Mbaye et al., 2019; Hou et al., 2021).

In a last step, the folding free energy values so obtained for each residue *i* were smoothed by taking a weighted average over the 5-residue sequence window [*i* − 2, *i* + 2] (Mbaye et al., 2019):

$${\bar{\Delta G}}_{\chi }^{i,\mu }=\frac{1}{1+2\gamma +2\beta }\left(\gamma \;\Delta G_{\chi }^{i-2,\mu }+\beta \;\Delta G_{\chi }^{i-1,\mu }+\Delta G_{\chi }^{i,\mu }+\beta \;\Delta G_{\chi }^{i+1,\mu }+\gamma \;\Delta G_{\chi }^{i+2,\mu }\right)$$ (7)

The weighting parameters *γ* and *β* were chosen to minimize the level of weaknesses in the membrane protein data set (see Section 2.5). For the N- and C-terminal residues, the smoothing was done on the residues present in the [*i* − 2, *i* + 2] sequence interval.

In this way, each residue *i* was tagged with two folding free energy values $\Delta G_{\chi }^{i,\text{EM}}$ and $\Delta G_{\chi }^{i,\text{TM}}$ for each statistical potential *χ*, irrespective of its location, in either EM or TM regions.

### 2.4 Membrane Propensity Index

We introduced a per-residue membrane propensity index MPr^*i*^ to predict to what extent a residue *i* in a folded protein corresponds to a stability strength or to a weakness when placed in a given, lipid or aqueous, environment. From a physico-chemical perspective, MPr^*i*^ estimates whether a residue shows a preference for the EM or TM environments, and can be interpreted as an index of stability of a residue within its structural context. It is defined as a linear combination of all folding free energy terms derived in the previous subsection:

$${\text{MPr}}^{i}=\underset{\mu }{\sum }\underset{\chi }{\sum }\alpha_{\chi }^{\mu }\;{\bar{\Delta G}}_{\chi }^{i,\mu }+\alpha_{L}logL+\alpha_{N}$$ (8)

where $\alpha_{\chi }^{\mu }$ , *α*_*L*_ and *α*_*N*_ are real-valued parameters that need to be optimized (see Section 2.5) and *L* is the protein length.

### 2.5 Model Training and Parameter Optimization

To estimate the membrane propensity index MPr^*i*^ introduced in Eq. 8, we needed to identify 42 free parameters introduced in Eqs 7, 8: *γ*, *β*, *α*_*L*_, *α*_*N*_ and 38 $\alpha_{\chi }^{\mu }$ parameters corresponding to one parameter for each of the 19 statistical potentials derived from either EM or TM regions (listed in Supplementary Table S1). We performed this parameter identification by minimizing the overall amount of structural weaknesses in the proteins from the $D_{\text{mem}}$ set. The general idea behind this procedure comes from the minimal frustration principle (Ferreiro et al., 2014) stating that proteins have evolved, and still evolve, to optimize the folding energy landscapes.

To perform the parameter identification on the $D_{\text{mem}}$ data set, we need to know if each of the residues in the proteins of this set is in an EM or TM region. For this, we searched the residue annotations in the OPM database (Lomize et al., 2012). These annotations were then assigned to the vector O , with the convention that zero corresponds to an EM region and one to a TM region. O is thus the target binary output in the model training. The data set is rather well balanced with about 40% of the residues belonging to TM regions and about 60% to EM regions. Note that the OPM annotations are predictions and may also suffer from inaccuracies.

We defined the cost function C as the square of the difference between the OPM annotations and the predicted membrane propensity MPr defined in Eq. 8:

$$C=\underset{i}{\sum }{\left({\text{MPr}}^{i}-O^{i}\right)}^{2}$$ (9)

where the sum is over all residues in the $D_{\text{mem}}$ training set. The parameter identification was done with Python (v.3) using the regression subroutine LinearRegression. Note that logistic regressions were also tested in the training process, but yielded less good results.

The performance of the predictor was evaluated using a strict leave-one-out cross validation procedure, in which each protein, in turn, was excluded from $D_{\text{mem}}$ and all steps of our computations, i.e., from the derivation of the statistical potentials to the computation of the per-site folding free energy and the parameter identification. The excluded protein was then predicted blindly. Note that, since the maximum pairwise sequence identity in $D_{\text{mem}}$ is 30%, the maximum identity between the training set proteins and the target protein is also 30%.

The BRANEart model outputs a continuous membrane propensity score MPr varying approximately between −0.5 and 1.5. Values that are close to one identify residues predicted to be stable in lipid environment, with the most stable having the highest MPr score. In contrast, MPr values close to zero represent residues that are predicted to be stable in aqueous environment, with the most stable having the lowest MPr score. Residues that strongly contribute to the stability of the region to which they belong are considered as stability strengths, and residues that would be more stable elsewhere in the protein are called stability weaknesses.

### 2.6 EM/TM Residue Classification

The membrane propensity score MPr was also used to set up a binary classifier that predicts whether residues in a membrane protein belong to TM or to EM regions. For that purpose, we transformed the continuous MPr scores into a discrete binary function, where 0 means EM and 1 TM, using an appropriate cutoff value *ϕ*_0_ such that a residue *i* is predicted to be in the TM region if MPr^*i*^ ≥ *ϕ*_0_ and in the EM region otherwise. The value of *ϕ*_0_ was identified to minimize the difference with OPM assignments.

To evaluate the performance of this classifier, we used the balanced accuracy (BACC) defined as:

$$\text{BACC}=\frac{1}{2}\left(\frac{\text{TP}}{\text{P}}+\frac{\text{TN}}{\text{N}}\right)$$ (10)

where TP and TN mean true positives and true negatives, respectively, and P an N positives and negatives.

In order to check our predictions using a threshold-independent metric, we also computed the AUC, i.e. the area under the receiver operating characteristic (ROC) curve, which plots sensitivity as a function of specificity for different threshold values.

### 2.7 Normalization of Crystallographic B-Factors

We considered crystallographic B-factors to get information about protein structural flexibility. However, B-factors of different protein structures cannot be compared without proper normalization (Smith et al., 2003). Two types of normalization were considered here.

The B-factors were extracted from the PDB files of all protein structures from $D_{\text{mem}}$ and $D_{\text{glob}}$ . For each residue, the average over the B-factors of the heavy main chain atoms was computed, and similarly for the heavy side chain atoms. In the next step, the outliers were removed from the distribution of the per-residue B-factors, separately for backbone and side chain, following the outlier removal technique introduced in Smith et al. (2003). The filtered sets of per-residue average B-factor values (*x*) were then normalized (*x*_*N*_) for each protein chain *P* in $D_{\text{mem}}$ and $D_{\text{glob}}$ using two standard techniques (Smith et al., 2003; Touw and Vriend, 2014):

1. The zero-mean-unit-variance technique: $x_{N}^{P}\left(i\right)=\left(x^{P}\left(i\right)-\mu^{P}\right)/\sigma^{P}$ , where *μ* ^*P*^ is the mean and *σ* ^*P*^ the standard deviation of *x*-values in protein *P*. The distribution of normalized $x_{N}^{P}$ values has zero mean and standard deviation equal to one.
2. The min-max scaling technique: $x_{N}^{P}\left(i\right)=\left(x^{P}\left(i\right)-\text{min}\left(x^{P}\right)\right)/\left(\text{max}\left(x^{P}\right)-\text{min}\left(x^{P}\right)\right)$ , with min and max meaning minimum and maximum. This technique scales *x* ^*P*^ to $x_{N}^{P}\in \left[0,1\right]$ .

## 3 Results and Discussion

### 3.1 Setting up BRANEart

We set up the BRANEart predictor, which predicts for each residue in a protein structure a membrane protein index MPr, defined as a linear combination of several types of statistical potential values, derived from either EM or TM regions of a set of membrane protein structures ( $D_{\text{mem}}^{\text{EM}}$ and $D_{\text{mem}}^{\text{TM}}$ ), as defined in Eqs 1–8. The coefficients of the linear combination were identified to follow OPM annotations at best (Lomize et al., 2012), through the minimization of Eq. 9, with the definition that an MPr close to one means a preference to be located in a TM region and an MPr close to zero, to be in an EM region. This identification was performed in strict cross validation (Section 2.5). In what follows, we only show the cross-validated values.

The MPr index yields a quantitative measure of the stabilizing or destabilizing contribution that a residue has in a specific environment, in other words, whether it acts as a weakness or a strength in that environment. Note that we compared here aqueous and lipid environments, but that this approach can be generalized to the comparison of other environments.

### 3.2 Large-Scale Application of BRANEart

We applied BRANEart to the membrane protein data set $D_{\text{mem}}$ and to the globular monomeric protein set $D_{\text{glob}}$ defined in Section 2.1, and computed the per-residue MPr score for the 56,715 and 1,258,648 amino acid residues contained in these two sets. Note that the huge difference in size between these two sets, of more than an order of magnitude, is due to the paucity of experimental structures available for membrane proteins.

As seen in Table 1, the mean ⟨MPr⟩ value is close to zero in globular proteins, ⟨MPr⟩ = 0.13, with a low standard deviation of *σ* = 0.13. This means that the large majority of the residues are stabler in an aqueous than in a lipid environment, which is obviously the case. Residues in membrane proteins have intermediate preferences (⟨MPr⟩ = 0.40) with a much larger *σ* of 0.37. However, splitting membrane proteins into TM and EM regions clarifies these findings: residues in EM regions clearly prefer to be in an aqueous environment (⟨MPr⟩ = 0.18), while residues inside the membrane prefer a lipid environment (⟨MPr⟩ = 0.74). These excellent results are a first validation of BRANEart.

**TABLE 1:** Mean per-residue index ⟨MPr⟩ and standard deviation *σ* of the distributions computed for the four protein structure sets considered in this paper; *N* is the number of residues in each data set.

Data set	Protein type	⟨MPr⟩	*σ*	*N*
$D_{\text{glob}}$	Globular proteins	0.13	0.13	1.3 × 10^6^
$D_{\text{mem}}$	Membrane proteins	0.40	0.37	5.7 × 10^5^
$D_{\text{mem}}^{\text{TM}}$	*Trans*-membrane regions	0.74	0.27	2.3 × 10^5^
$D_{\text{mem}}^{\text{EM}}$	Extra-membrane regions	0.18	0.22	3.4 × 10^5^

**FIGURE 1:**
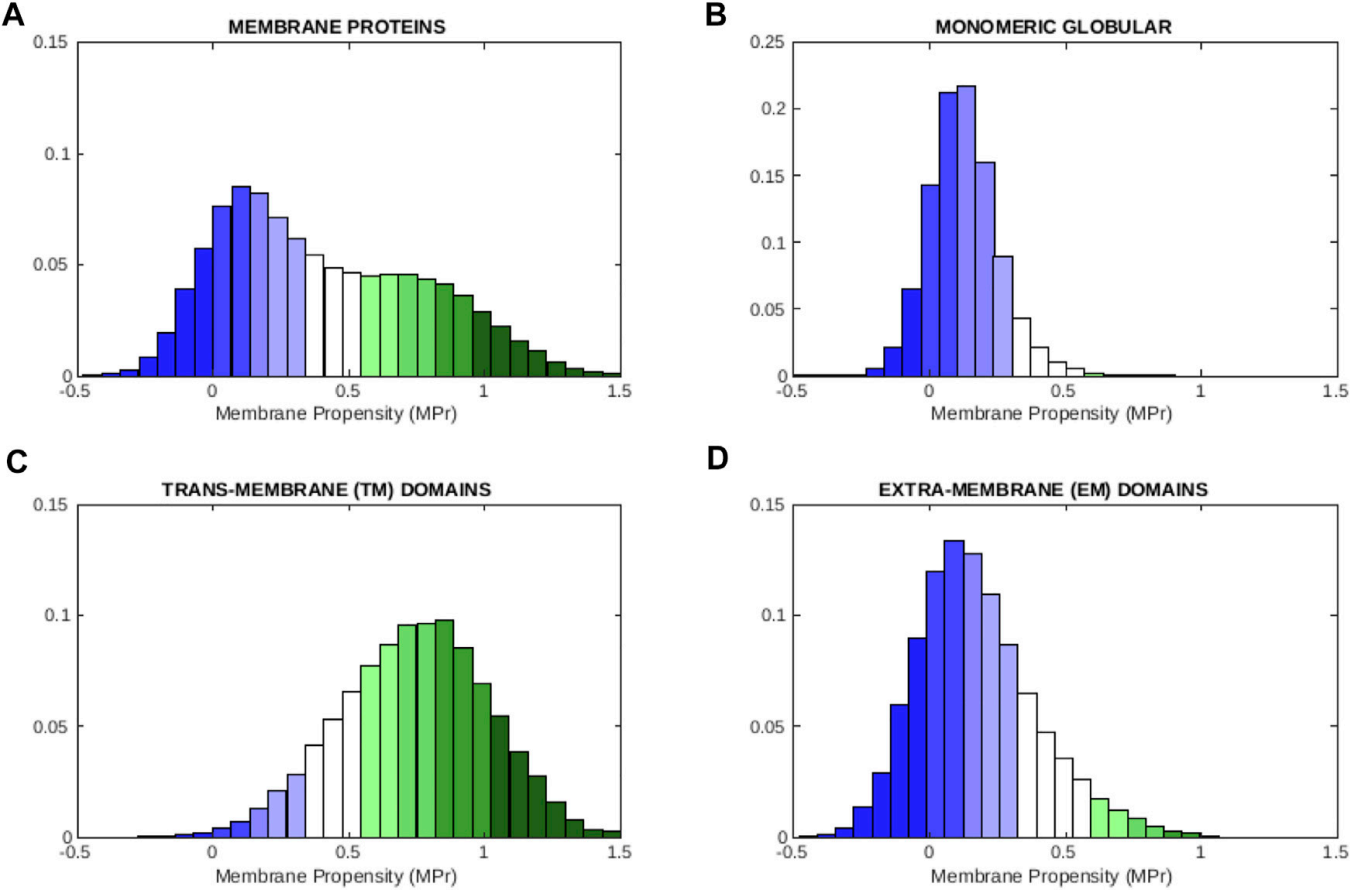
Distribution of MPr scores computed from different of protein data sets: **(A)** membrane proteins $D_{\text{mem}}$ , **(B)** globular proteins $D_{\text{glob}}$ , **(C)** TM regions $D_{\text{mem}}^{\text{TM}}$ and **(D)** EM regions $D_{\text{mem}}^{\text{EM}}$ . The color code is defined in Table 2.

The MPr distributions are plotted in Figure 1 for each of the four data sets. They all show a unimodal bell-shaped distribution, except the one computed from $D_{\text{mem}}$ which is more spread out with two peaks, a narrow peak and a flatter one. This distribution is the union of the two unimodal distributions computed from the sets $D_{\text{mem}}^{\text{TM}}$ and $D_{\text{mem}}^{\text{EM}}$ , which are shifted relative to each other and show a marked preference for lipid and aqueous environments, respectively. The distribution from $D_{\text{mem}}^{\text{EM}}$ is roughly centered around the same MPr value as the one obtained from $D_{\text{glob}}$ , but is less peaked although the proteins from both sets are in an aqueous environment. This suggests that there are more stability weaknesses in membrane proteins than in globular proteins or more precisely, that there are more residues in the EM regions of membrane proteins than in globular proteins which would prefer to be in a lipid environment.

To graphically visualize the level of stability of a residue, we defined stability classes in terms of the *ϕ*_0_ threshold value defined in section 2.6 and the standard deviation of the MPr distribution and we colored the residues accordingly, as explained in Table 2. Residues that are strong in aqueous environments but weak in lipid environments are colored in blue. Residues that are strong in the phosholipid bilayer and weak in aqueous solvent are colored in green. Different color graduations were defined to further differentiate between highly stable, stable, moderately stable and mildly stable residues in EM regions, and equivalently in TM regions.

**TABLE 2:** Classes of MPr scores defined in terms of the classification threshold *ϕ*_0_ defined in section 2.6 and the standard deviations *σ*™ and *σ* ^EM^ of the MPr distributions computed from $D_{\text{mem}}^{\text{TM}}$ and $D_{\text{mem}}^{\text{EM}}$ , respectively. The colors used in the visualization frameworks of the BRANEart web server are here defined.

Color	Index range	Type

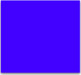	MPr^*i*^ ≤ *ϕ*_0_ − 2*σ* ^EM^	Highly stable in water
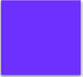	*ϕ*_0_ − 2*σ* ^EM^ < MPr^*i*^ ≤ *ϕ*_0_ − 3/2*σ* ^EM^	Stable in water
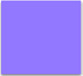	*ϕ*_0_ − 3/2*σ* ^EM^ < MPr^*i*^ ≤ *ϕ*_0_ − *σ* ^EM^	Moderately stable in water
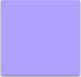	*ϕ*_0_ − *σ* ^EM^ < MPr^*i*^ ≤ *ϕ*_0_ − 1/2*σ* ^EM^	Mildly stable in water
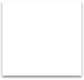	*ϕ*_0_ − 1/2*σ* ^EM^ < *MPr* ^*i*^ ≤ *ϕ*_0_ + 1/2*σ* ^TM^	Neutral
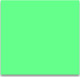	*ϕ*_0_ + 1/2*σ* ^TM^ < MPr^*i*^ ≤ *ϕ*_0_ + *σ* ^TM^	Mildly stable in lipids
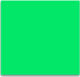	*ϕ*_0_ + *σ* ^TM^ < MPr^*i*^ ≤ *ϕ*_0_ + 3/2*σ* ^TM^	Moderately stable in lipids
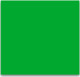	*ϕ*_0_ + 3/2*σ* ^TM^ < MPr^*i*^ ≤ *ϕ*_0_ + 2*σ* ^TM^	Stable in lipids
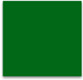	*ϕ*_0_ + 2*σ* ^TM^ ≤ MPr^*i*^	Highly stable in lipids

Interestingly, the amount of strengths and weaknesses in TM and EM regions are almost identical, as computed from the MPr distributions: 7% of the residues are weak in both regions, 75% are strong and the remaining ones are neutral. Without surprise, most residues thus contribute to the stabilization of the regions to which they belong; note that the functional residues are usually among the weak residues.

**FIGURE 2:**
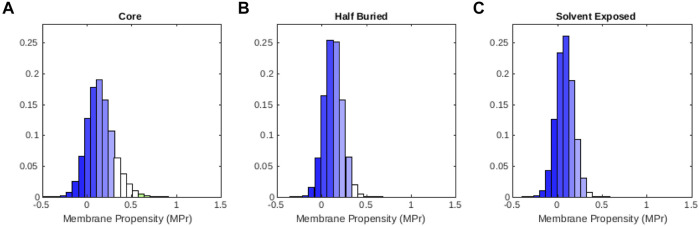
Distribution of MPr scores computed from the globular protein set $D_{\text{glob}}$ for residues that are **(A)** situated in the core, **(B)** half buried and **(C)** solvent exposed. The color code is defined in Table 2.

We also examined how the MPr values change as a function of the solvent accessibility in globular proteins. Therefore, we divided all the residues in $D_{\text{glob}}$ into three disjoint bins: core residues with solvent accessibility < 20%, intermediate residues with 20% ≤ accessibility < 50%, and surface residues with accessibility ≥50%. The average MPr score in these groups gradually drops from core to surface: 0.15 (*σ* = 0.15), 0.13 (*σ* = 0.10), 0.08 (*σ* = 0.10). As seen in Figure 2, the whole MPr distribution is shifted towards lower values, and becomes more peaked. This reflects the fact that residues in the core of globular proteins locally feel a hydrophobic environment and have a slightly higher MPr, while surface residues are in direct contact with water and have a lower MPr.

### 3.3 Application to *α*-helical and *β*-barrel Membrane Proteins

Membrane proteins of which the TM region has an *α*-helical or a *β*-barrel conformation exhibit different folding and stability properties (Leman et al., 2018; Mbaye et al., 2019). Indeed, the former type of proteins are essentially localized in the cytoplasmic membranes of eukaryotic and prokaryotic cells and quite rarely in outer membranes, whereas the latter type of proteins is found in outer membranes of Gram-negative bacteria, mitochondria or chloroplasts.

**FIGURE 3:**
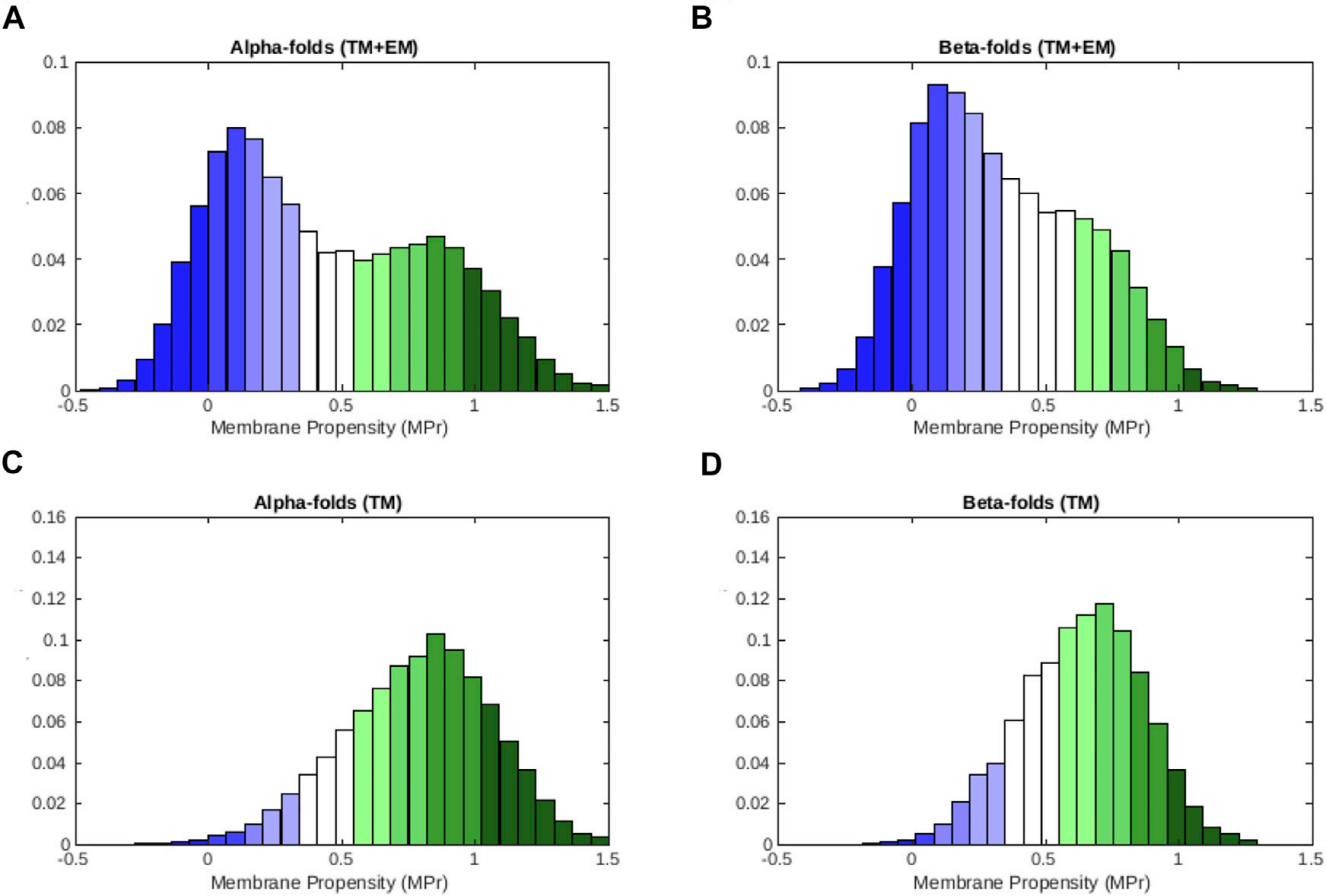
MPr distribution computed from the membrane proteins in $D_{\text{mem}}$ that have an *α*-helical or *β*-barrel structure. **(A)** *α*-folds (TM and EM), **(B)** *β*-folds (TM and EM), **(C)** TM region of *α*-folds and **(D)** TM region of *β*-folds. The color code is defined in Table 2.

We observe from the distributions depicted in Figures 3A,B that residues pertaining to *α*-helical folds have a bigger preference for lipid environments than those pertaining to *β*-barrel folds. Indeed, average ⟨MPr⟩ values are equal to 0.44 (*σ* = 0.40) and 0.34 (*σ* = 0.31) for *α* and *β* proteins, respectively. If we focus on the TM regions, this tendency is even more marked (Figures 3C,D): ⟨MPr⟩ = 0.78 (*σ* = 0.28) and 0.63 (*σ* = 0.23).

These results are in line with a series of facts. First, *α*-helices are coiled structures stabilized by regularly spaced hydrogen bonds between residues at positions *i* and *i* + 4 along the polypeptide chain. In contrast, *β*-sheets are stabilized by hydrogen bonds between residues from different *β*-strands, which are usually not close along the chain (Berg et al., 2002). They thus harbour greater geometric variability than *α*-helices and display greater deviations from ideal backbone bond angles (Touw and Vriend, 2010; Basu et al., 2014). Secondly, *β*-fold membrane proteins mainly have channel or porin conformations, through which molecules can cross the membrane. The internal faces of these *β*-barrels are therefore more hydrophilic even though they are in TM regions. They thus often correspond to weaknesses when computed in lipid environments. Finally, the asymmetrical bilayer of phospholipids in which *β*-barrel proteins are usually inserted have different characteristics than standard phospholipid bilayers. This implies that our statistical potentials generated from TM regions of the whole set of membrane proteins could be less accurate for this kind of proteins (see Mbaye et al. (2019) for further details).

### 3.4 Protein Embedding in the Membrane

The MPr score can be used not only to identify weak and strong regions but also to predict the protein embedding in the lipid bilayer membrane and more specifically, whether residues are inside or outside the membrane. For that purpose, we transformed the per-residue MPr scores into a binary function, where 0 and 1 mean EM and TM, respectively, using the *ϕ*_0_ threshold value defined in section 2.6. We computed the BACC and AUC scores (Eq. 10 and Supplementary Material S4) between the so predicted membrane assignments and OPM annotations, in a strict protein-level cross validation (Section 2.5). The BACC score is equal to 0.87 and the AUC score reaches 0.94.

These scores are very high and we did not expect better results. Indeed, weak residues in TM and EM regions fall in the class of wrong predictions, even though some of them are truly weak and are essential for membrane protein functioning as we will see in the next two subsections.

Note that we used annotations of OPM (Lomize et al., 2012) to train the predictor, and that these annotations are actually also computational predictions. Some discrepancies between the results of our prediction and OPM could therefore be due to errors of OPM rather than of our predictor. Moreover, the discrepancies between OPM and BRANEart predictions are essentially located close to the membrane borders and highlight some structural flexibility in these regions as illustrated in Supplementary Figure S5. We could use our predictor to yield new EM/TM assignments, which would differ from OPM assignments in some places. Alternatively, the MPr score could be used as a feature, in combination with other features, to improve the accuracy of the membrane protein embedding in the membrane (Lomize and Pogozheva, 2013; Postic et al., 2016).

### 3.5 Probing Membrane Helix Associations

Experimental data about the association energy $\Delta G_{\alpha }^{ass}$ of a series of 16 membrane structures that contain a single *α*-helix crossing the membrane were collected from Lomize et al. (2020). We analyzed how the MPr score of these transmembrane structures change upon dimerization or tetramerization and whether there is a quantitative relation between this score and the experimental $\Delta G_{\alpha }^{ass}$ values. For this purpose, we defined the quantity T as the difference in MPr score between the transmembrane helix dimer *AB* and the two helices *A* and *B*:

$$T=\frac{1}{2}\left(\overset{L_{A}+L_{B}}{\underset{i}{\sum }}{\text{MPr}}_{i}\left(AB\right)-\overset{L_{A}}{\underset{i}{\sum }}{\text{MPr}}_{i}\left(A\right)-\overset{L_{B}}{\underset{i}{\sum }}{\text{MPr}}_{i}\left(B\right)\right)$$ (11)

where *L*_*A*_ and *L*_*B*_ are the number of residues in *A* and *B*. In the case the complex is a tetramer, the MPr score of the four separate monomers are subtracted from that of the complex, and divided by four. We computed the Pearson correlation coefficient *r* between $\Delta G_{\alpha }^{ass}$ and T for the 16 transmembrane helical complexes, and found a very high value of 0.91 (*p*-value $<10^{-5}$ ).

For comparison, we computed the correlation coefficient between $\Delta G_{\alpha }^{ass}$ and the interaction energy predicted by the popular tools TMPFold (Lomize et al., 2020), PDBePISA (Krissinel and Henrick, 2007), and PRODIGY (Xue et al., 2016). We found these correlation coefficients to be equal to 0.89, 0.85 0.75, respectively. BRANEart thus outperforms these three methods on the considered test set.

Note that BRANEart was not designed for predicting stability inside the membrane, but rather to detect the protein regions that prefer to be inside or outside the membrane. This result comes thus as an additional, unexpected, application of BRANEart. Note also that the predictions were completely blind as no experimental association energies were used in the model construction.

The list of all proteins with their experimental $\Delta G_{\alpha }^{ass}$ values, the T score and the predictions of TMPFold, PDBePISA and PRODIGY can be found in the github repository: https://github.com/3BioCompBio/BRANEart.

### 3.6 Relating the MPr Score to Biophysical Quantities

This section explores the relationships between the MPr score that identifies stability strength and weakness regions in membrane proteins and several quantities describing structural and biophysical residue properties. We started by computing the correlation between MPr and different hydrophobicity scales for all residues belonging to $D_{\text{mem}}^{\text{TM}}$ . A wide variety of hydrophobicity scales have been derived in the past decades using experimental or knowledge-based approaches, which describe the difference in stability of residues embedded in water or lipid bilayers (Engelman et al., 1986; Hessa et al., 2005; Koehler et al., 2009; Moon and Fleming, 2011). These scales are employed in algorithms that predict the TM segments of membrane proteins (Deber et al., 2001; Tian et al., 2020).

Our BRANEart MPr score is only weakly correlated with these various hydrophobicity scales: the Pearson correlation coefficient is always around *r* =−0.20 (see Supplementary Material S3.1 for details). This is not surprising as the MPr score contains much more information than simple hydrophobicity values and takes into account structural data and their complexity. It classifies TM and EM residues with much higher accuracy and can properly describe the local stability of different conformations of the same membrane protein, as shown in the next subsection.

We also explored the relationship between the BRANEart MPr score and the depth in the lipid bilayer. Our membrane-dependent potentials are based on a series of conformational descriptors and their combinations, but do not use depth in contrast to some other statistical potentials (Senes et al., 2007; Schramm et al., 2012). Despite this, we found a good correlation between the per-residue MPr scores and the absolute values of the Z-depth, defined as the distance between the residue side chain centroids and the plane parallel to the membranes cutting the bilayer into two equal parts. The Pearson correlation coefficient is indeed *r* =−0.55. This means that the larger the MPr score and thus the larger the preference of a residue for a lipid environment, the deeper it is embedded in the lipid membrane. Note that the relationship is non-linear as can be seen from Supplementary Figure S3. This suggests the use of non-linear functions of our statistical potentials to predict the Z-depth more precisely.

Finally, we investigated whether MPr scores are correlated with residue flexibility, estimated from crystallographic B-factors that indicate the relative vibrational motion of atoms in different parts of the structure. To be able to compare the B-factors of different proteins, they need to be properly normalized. We used two normalization techniques, the zero-mean-unit-variance and min-max-scaling techniques, described in Methods subsection 2.7. We computed the Pearson correlation coefficient between the MPr scores and the per-residue normalized B-factors of all residues in $D_{\text{mem}}$ , separately for backbone and side chains. The correlations are all low and negative, with *r* in the [−0.1, −0.3] range, both for side chains and main chains, for the two normalization schemes, and for TM and EM regions. For comparison, we also computed the correlation between MPr and B-factors in the globular protein set, $D_{\text{glob}}$ , with very similar results. Further details can be found in Supplementary Material S3.3.

This characteristic anti-correlation can be explained by the opposite trends of MPr scores and B-factors. For the B-factor, the higher the value, the higher the degree of flexibility. For the MPr score, a higher value means a higher preference for lipid environments that restrict atomic movements. The anti-correlation thus means that EM regions are more flexible than TM regions. The low value of the correlation is due to the fact that the MPr score contains information about stability across different environments, which is much more than information about flexibility. Note, moreover, that stability and MPr are related to free energy in which flexibility is entropically favorable.

### 3.7 Application to Leucine Transporters

In general, membrane proteins undergo conformational changes to accomplish their biological functions (Latorraca et al., 2017; Chataigner et al., 2020). Residues that are at the basis of these large-scale movements are often stability weaknesses (Hou et al., 2021) or in other words, frustrated (Ferreiro et al., 2014). Indeed, functional constraints prevent them to adopt conformations with high stability contributions. Here we show how we can use the MPr score to gain insight into the functional role of these residues. Note that, to our knowledge, no other method employs dedicated energy functions to study frustration in membrane proteins.

As an example, we analyzed leucine transporter (LeuT), a bacterial homodimeric protein containing twelve transmembrane helices, which uses the electrochemical potential of sodium ions to transport leucine from outside to inside the cell (Penmatsa and Gouaux, 2014). Both the substrate-free outward-open LeuT structure and the inward-open apo conformation have been resolved *via* X-ray crystallography (Krishnamurthy and Gouaux, 2012) (PDB codes: 3tt1 and 3tt3, respectively).

**FIGURE 4:**
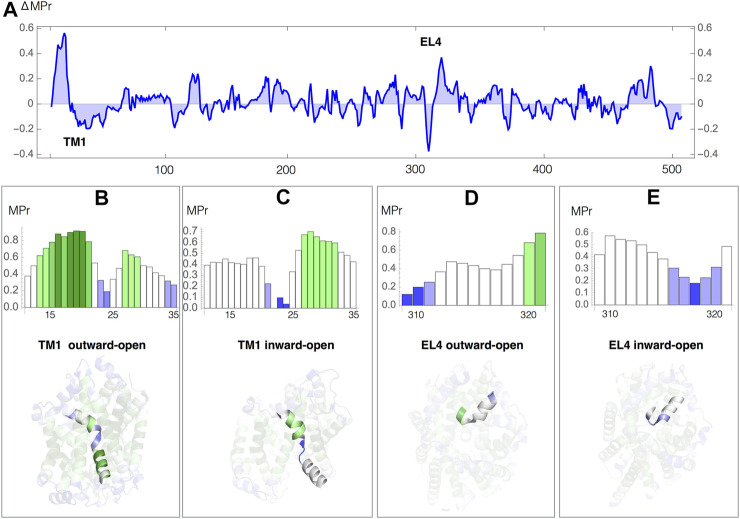
Leu transporter: MPr score in the outward-open (PDB code 3tt1) and inward-open (PDB code 3tt3) conformations **(A)** Difference in MPr score (ΔMpr) between the two conformations as a function of the sequence position; the positions of the helices TM1 and EL4 are indicated. **(B–E)** MPr of the TM1 and EL4 helices as a function of the sequence position, and TM1 and EL4 helices embedded in the 3D structure, colored according to the code defined in Table 2; **(B)** TM1, outward-open conformation; **(C)** TM1, inward-open conformation; **(D)** EL4, outward-open conformation; **(D)** EL4, inward-open conformation.

We analyzed the difference in membrane propensity index (ΔMPr) between the two conformations in Figure 4A. We can easily identify the two regions with highest ΔMPr in absolute value: the transmembrane helix 1 (TM1) and the extracellular helix 4 (EL4), which are key elements that allow the opening and closing of the intracellular and extracellular gates, respectively (Krishnamurthy and Gouaux, 2012). We thus focused on these two regions and analyzed their MPr values in both outward and inward conformations (Figures 4B–E).

The transmembrane segment TM1 (residues 11–35) is a kinked helix formed by two helical regions, TM1a (residues 11–21) and TM1b (residues 25–35), separated by a hinge fragment (residues 22–24). TM1a undergoes a large movement upon opening of the intracellular gate (Figures 4B,C). Despite the fact that the whole TM1 is inside the lipid membrane, the hinge residues have a very low MPr score in both conformations. They are thus stability weaknesses, relating them to their functional role in facilitating the opening and closing of the gate, as confirmed by experiments (Krishnamurthy and Gouaux, 2012). The MPr of TM1a changes significantly during the (closed → open) conformational transition. In the inward-open conformation, when the intracellular gate is open, TM1 is only found weakly stable (Figure 4C), whereas in the outward-open conformation, when the intracellular gate is closed, TM1a establishes stabilizing contacts with TM5 and TM7 which is reflected by high MPr values (Figure 4B).

In a similar way, we identified the strong and weak residues in the outer membrane environment related to the conformational movement that EL4 undergoes while opening and closing of the extracellular gate. EL4 (residues 309–321) is a helix of which the last residues (319–321) are unwound and called hinge; its position appears displaced in the outward-open and inward-open conformations. In the outward-open conformation, when the extracellular gate is open, the N-terminal EL4 residues 309–311 are stable in aqueous environment but the hinge residues are weak (Figure 4D). While closing the gate, EL4 changes in terms of its stability profile, with its N-terminus becoming weak and its C-terminus and the hinge getting more stable (Figure 4C) due to extensive contacts including hydrophobic interactions and a hydrogen bond between Ala 319 and Asp 401 in TM10.

This example illustrates how we can use BRANEart to identify in a simple way residues in membrane proteins that are not optimized for their stability, which is particularly relevant for functional residues.

### 3.8 Application to Human Phospholamban

**FIGURE 5:**
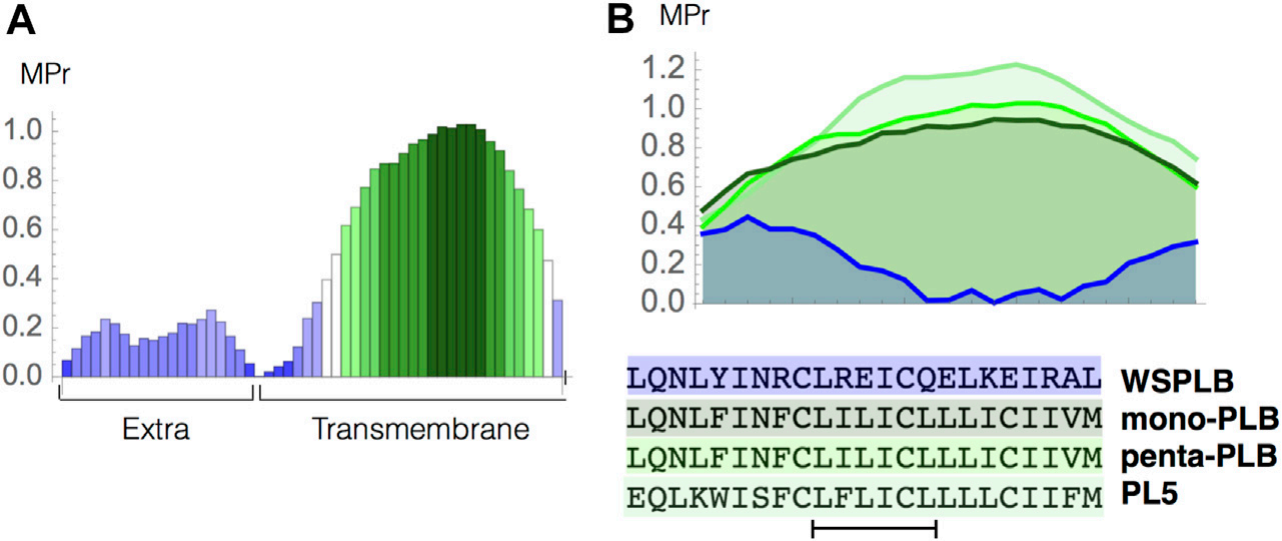
Phospholamban case study. **(A)** MPr values computed for the pentameric structure of the full-length phospholambam (PDB code 1zll); **(B)** amino acid sequences and MPr values for the TM fragment (residues 28–50) of four different PLB structures: PLB monomer (light green, PDB code 1zll), PLB pentamer (green, PDB code 1zll), redesigned pentamer PL5 (dark green, PDB code 6mqu) and water soluble PLB tetramer (blue, PDB code 1yod).

As a second test case, we studied the stability of human phospholamban (PLB), a protein anchored into the cardiac sarcoplasmic reticulum membrane, which is essential to myocardial contractility (Koss and Kranias, 1996). Let us start with the analysis of the MPr index as a function of the sequence, computed from the standard pentameric form of PLB (PDB code 1zll). As shown in Figure 5A, the two domains of the protein are easy to identify: the EM part (residues 1–22) has low MPr values and thus the propensity to be stable in water, and the TM part (residues 23–52) has overall much higher MPr values indicating its stability in lipids. A closer look shows that the TM region must be divided in two: the C-terminal part (residues 33–52) is apolar, contains the well known motif LxxIxxx (Mravic et al., 2019) and has high MPr values indicating its stability in lipids, whereas the N-terminus (residues 23–32) is a TM weakness.

Note that the latter part connects the EM and TM regions and is very close to the water-membrane interface. It thus probably changes dynamically from the lipid TM to the aqueous EM environment and *vice versa*. This prediction is in perfect agreement with both NMR data (Oxenoid and Chou, 2005), molecular dynamics simulations (Kim et al., 2009; Mravic et al., 2019) and mutagenesis data (Fujii et al., 1989; Simmerman et al., 1996), which identified this region as highly dynamical, unimportant for the stabilization of the structure, but crucial for modulating functional interactions of PLB.

We also analyzed the MPr index of the different types of PLB structures. More specifically, we compared the stability of the wild-type monomeric and pentameric forms (PDB code 1zll) (Oxenoid and Chou, 2005), of a highly stable designed pentameric variant (PL5, PDB code 6mqu) (Mravic et al., 2019) and of a water-soluble tetrameric variant (WSPLB, PDB code 1yod) (Slovic et al., 2005). The MPr values of the TM region (residues 28–50) in all these structures are reported in Figure 5B. The TM region of the monomeric form shows a clear preference for the lipid environment with all residues having an MPr value bigger than ∼ 0.5. The pentamer form is predicted to be more stable than the monomer form since inter-chain interactions between the TM fragments strongly stabilize this structural assembly. Note that both the monomer and pentamer forms do exist in phospholipid bilayers even though the latter is dominant. Such oligomer equilibrium is dynamic and can change due to mutations or phosphorylation (Cornea et al., 1997).

The PL5 variant has the largest MPr values and is the most stable form in the lipid environment. This is again in perfect agreement with experiments; in redesigning this protein fragment, the polar residues have been substituted by apolar residues to get stronger interchain interactions driven by apolar sidechain packing and an increased stability of the overall fold (Mravic et al., 2019). Finally, the redesigned, truncated, water-soluble variant has extremely low propensities to be in a lipid environment, as can easily be seen from our predictions.

### 3.9 Application in the Evaluation of Docked Complexes

BRANEart has recently been applied in the context of COVID-19 research to cross-check the correctness of docked peptide-protein complexes between angiotensin II and its cognate receptor, angiotensin converting enzyme 2 (ACE2) (Basu et al., 2021). The survey was performed by comparing the MPr profiles of the peptide ligand and the docking sites in the top-ranked docked poses returned by ClusPro 2.0 (Desta et al., 2020). BRANEart unanimously selected peptides mapping to ACE2’s deep pocket buried in the hydrophobic core. This result shows the usefulness of BRANEart to analyze hydrophobicity compatibility between protein and/or peptide partners.

### 3.10 BRANEart Web Server: Features and Functionalities

We implemented our structure-based prediction method into the easy-to-use BRANEart web server. To run a query, the user first chooses the structure of a membrane protein by either providing its 4-letter code which is then automatically retrieved if available in the PDB, or by uploading a protein structure file in PDB format. The user is then asked to select the relevant chain(s) on which BRANEart has to perform the predictions, taking into account the other chains present in the structure file. Finally, the computation starts.

The main BRANEart output is the MPr score for each amino acid residue in the selected chain(s) of the target protein structure. In addition to returning these values as a downloadable text file, the web server also displays a table with the MPr of each residue of the targeted protein chain(s), colored according to the code defined in Table 2. In addition, BRANEart provides a multi-featured browser tool to visualize the protein 3D structure, where each residue is colored according to its MPr score. It has several advanced visualization functionalities. More precisely, it is possible to:

• hide/show specific chain(s) in the displayed structure, and zoom in and out by left dragging the mouse.
• switch to a full screen visualization mode and take a “.png” snapshot of the visualized structures.
• select a residue of interest by double clicking on it. The neighboring residues are then displayed in “ball and sticks”.
• download a PyMol session file (.pml) (The PyMOL Molecular Graphics System, Version 2.0 Schrödinger, LLC) to switch to the PyMol representation of the 3D structure.

For further technical details and information about the web server, we refer to the BRANEart help page.

## 4 Conclusion

We presented BRANEart, a computational method to identify the stability strengths and weaknesses in membrane proteins. Extending and combining the newly developed membrane statistical potentials introduced in Mbaye et al. (2019), we defined a MPr score that quantifies whether a residue is stable in a lipid or aqueous environment. Large-scale predictions and applications to test cases show BRANEart’s ability to correctly identify regions in their respective environment that strongly contribute to the stabilization of their host protein, and residues that have instead low impact on stabilization but have functional roles.

Note that our approach can be extended to identify strengths and weaknesses in other environments than the membrane, for example in hot and cold environments using temperature-dependent potentials (Folch et al., 2010).

We additionally provided a user friendly web server that, on the basis of the 3D structure of the target membrane protein, computes the MPr score for each residue in the input structure. Visualization tools are provided which simplify the understanding and interpretation of BRANEart results.

To our knowledge, BRANEart is the first accessible, fast and accurate tool that use dedicated membrane protein potentials to identify stability strengths and weaknesses in membrane proteins. The use of such mean force potentials drastically simplifies the analysis of membrane protein stability, since the effect of the lipid environment is considered in an implicit manner. BRANEart can be used in a wide series of applications that range from the analysis of the conformational changes in membrane proteins, the embedding of proteins in the lipid membrane, to the identification of residues to target for the rational modification of biophysical characteristics of membrane proteins.

## Data Availability

The original contributions presented in the study are included in the article/Supplementary Material, further inquiries can be directed to the corresponding authors.
