# Supplementary material for: BRANEart: Identify Stability Strength and Weakness Regions in Membrane Proteins
Source: Front Bioinform. 2021 Dec 2;1:742843. doi: 10.3389/fbinf.2021.742843 (PMC9581023; doi:10.3389/fbinf.2021.742843)
Supplement: Supplementary file 1 [file DataSheet1.PDF]

Supplementary Material

BRANEart: identify stability strength and weakness regions in membrane proteins

Sankar Basu, Simon S. Assaf, Fabian Teheux, Marianne Rooman and Fabrizio Pucci

Index

S1. Statistical potentials and per-residue folding free energies used in this study.

S2. Details on the per-residue folding free energy construction.

S3. Relation between strength and weakness regions and biophysical quantities.

S4. EM/TM residue classification.

S5. Embedding in the lipid membrane: BRANEart versus OPM.

S1. Statistical potentials and per-residue folding free energies used in this study

We considered in this study 19 different statistical potentials ΔW, defined in Eqs (1)-(2) of the main manuscript, and listed in Table S1. For each type of potential, we computed the contribution to the folding free energy ΔG^i of each residue i in a protein structure, using Eqs (3)-(7) of the main manuscript; some details on this construction can be found in the next section (Section S2).

Per-residue Energy	Potential	χ	sequence window	Dataset (μ)	Type
$\Delta G^{i,\mu}_{ss}$	ΔW^{μ}_{ss}	$s_i s_j$	$\lvert i-j\rvert$ <8	EM ∨ TM	Local
$\Delta G^{i,\mu}_{tt}$	ΔW^{μ}_{tt}	$t_i t_j$	$\lvert i-j\rvert$ <8	EM ∨ TM	Local
$\Delta G^{i,\mu}_{aa}$	ΔW^{μ}_{aa}	$a_i a_j$	$\lvert i-j\rvert$ <8	EM ∨ TM	Local
$\Delta G^{i,\mu}_{ta}$	ΔW^{μ}_{ta}	$t_i a_j$	$\lvert i-j\rvert$ <8	EM ∨ TM	Local
$\Delta G^{i,\mu}_{st}$	ΔW^{μ}_{st}	$s_i t_j$	$\lvert i-j\rvert$ <8	EM ∨ TM	Local
$\Delta G^{i,\mu}_{sa}$	ΔW^{μ}_{sa}	$s_i a_j$	$\lvert i-j\rvert$ <8	EM ∨ TM	Local
$\Delta G^{i,\mu}_{sst}$	ΔW^{μ}_{sst}	$s_i s_j t_k$	$k-8 \leq i < j \leq k+8$	EM ∨ TM	Local
$\Delta G^{i,\mu}_{stt}$	ΔW^{μ}_{stt}	$s_i t_j t_k$	$k-8 \leq i < j \leq k+8$	EM ∨ TM	Local
$\Delta G^{i,\mu}_{ssa}$	ΔW^{μ}_{ssa}	$s_i s_j a_k$	$k-8 \leq i < j \leq k+8$	EM ∨ TM	Local
$\Delta G^{i,\mu}_{saa}$	ΔW^{μ}_{saa}	$s_i a_j a_k$	$k-8 \leq i < j \leq k+8$	EM ∨ TM	Local
$\Delta G^{i,\mu}_{sta}$	ΔW^{μ}_{sta}	$s_i t_j a_k$	$k-8 \leq i < j \leq k+8$	EM ∨ TM	Local
$\Delta G^{i,\mu}_{sd}$	ΔW^{μ}_{sd}	$s_i d_{ij}$	$i \neq j, j+1$	EM ∨ TM	Distance
$\Delta G^{i,\mu}_{ad}$	ΔW^{μ}_{ad}	$a_i d_{ij}$	$i \neq j, j+1$	EM ∨ TM	Distance
$\Delta G^{i,\mu}_{td}$	ΔW^{μ}_{td}	$t_i d_{ij}$	$i \neq j, j+1$	EM ∨ TM	Distance
$\Delta G^{i,\mu}_{sad}$	ΔW^{μ}_{sad}	$s_i a_j d_{ij}$	$i \neq j, j+1$	EM ∨ TM	Distance
$\Delta G^{i,\mu}_{sds}$	ΔW^{μ}_{sds}	$s_i d_{ij} s_j$	$i \neq j, j+1$	EM ∨ TM	Distance
$\Delta G^{i,\mu}_{std}$	ΔW^{μ}_{std}	$s_i t_j d_{ij}$	$i \neq j, j+1$	EM ∨ TM	Distance
$\Delta G^{i,\mu}_{ada}$	ΔW^{μ}_{ada}	$a_i d_{ij} a_j$	$i \neq j, j+1$	EM ∨ TM	Distance
$\Delta G^{i,\mu}_{tdt}$	ΔW^{μ}_{tdt}	$t_i d_{ij} t_j$	$i \neq j, j+1$	EM ∨ TM	Distance

Table S1. List of per-residue folding free energy contributions and corresponding type of statistical potentials used in this study, with their characteristics.

The potentials are characterized by different sequence and structural descriptors s, t, a and d: s is an amino acid type, t, one of the seven (ϕ, ψ, ω) backbone torsion angle domains defined in [1], and a, a solvent accessibility bin where the solvent accessibility is defined as the ratio (in %) between the solvent accessible surface area of a residue in the structure and in an extended Gly-X-Gly conformation [2, 3]. Five discrete bins of solvent accessibility were considered: 0 to 5%, 5 to 15%, 15 to 30%, 30 to 50% and 50 to 100%. Finally, d is defined as the spatial distance between the average side chain geometric centers of two residues separated by at least one residue along the polypeptide chain [2]. The distance values between 3.0 to 8.0 Å were divided into 25 discrete bins of 0.25 Å width, with an additional bin containing all distances greater than 8.0 Å and another bin containing all distances smaller than 3 Å.

The potentials that include 'd' as structural descriptor are called distance potentials and describe tertiary interactions. The residues i and j they involve are separated by a least one

residue along the chain. The other potentials are called local potentials, as they involve residues (i, j) or (i, j, k) that are in a sequence window of ± 8 residues around the central residue i. They describe local interactions along the polypeptide chain.

Each of these potentials was derived separately from the datasets $\mathcal{D}^{\rm TM}_{\rm mem}$ and $\mathcal{D}^{\rm EM}_{\rm mem}$ containing either TM or EM regions of membrane proteins. For further technical details about our implementation of statistical potentials, we refer to our previous articles [4, 5, 6].

S2. Details on the per-residue folding free energy construction

We show in Fig. S1 two examples of distribution of folding free energy over different residues.

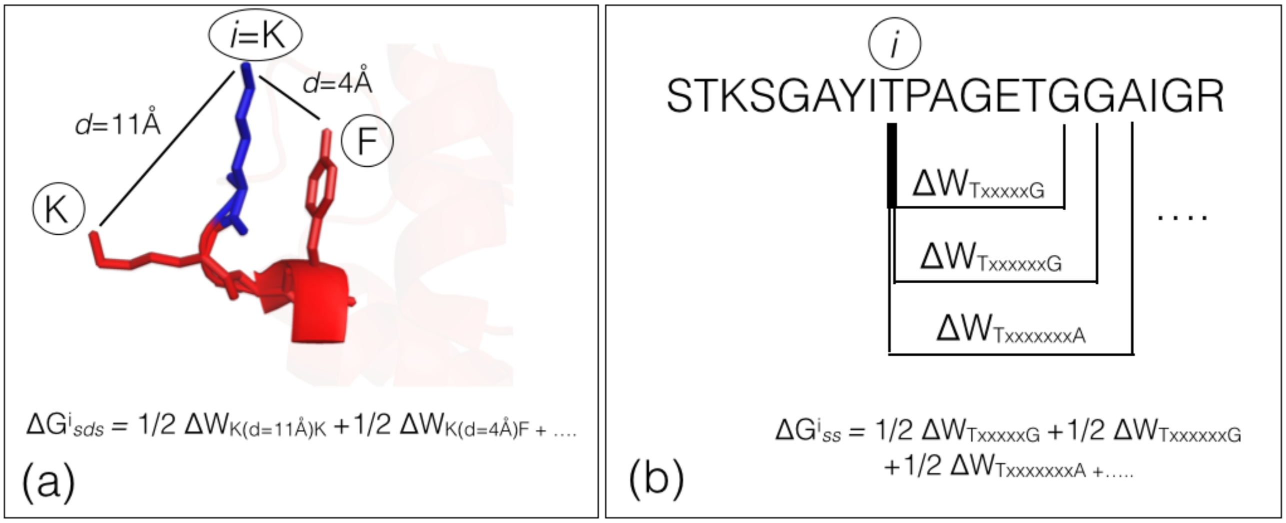

Figure S1. Schematic representation of the way in which the folding free energy contributions ΔG^i for each residue i in a protein structure are defined in terms of the corresponding statistical potentials ΔW. The general ΔG^i definition is given in Eqs (3)-(7) of the main manuscript; the statistical potentials ΔW are defined in Eqs (1)-(2) of the main manuscript and details are given in Section S1; 'x' denotes any residue. Construction of: (a) $\Delta G^{i,\mu}_{sds}$; (b) $\Delta G^{i,\mu}_{ss}$.

S3. Relation between strength and weakness regions and biophysical quantities.

S3.1 Relation between MPr score and hydrophobicity scales

We analyzed the correlation between MPr scores and hydrophobicity scales derived either from experimental data or using knowledge-base approaches [7, 8, 9, 10]. The Pearson correlation coefficients are reported in Table S2. They are clearly very low.

Hydrophobicity	r
[7]	-0.19
[8]	-0.15
[9]	-0.19
[10]	-0.17

Table S2. Pearson correlation coefficient r between the per-residue MPr scores and hydrophobicity values according to different scales described in the cited articles. The correlation is computed for all residues in the $\mathcal{D}_{\rm mem}^{\rm TM}$ data set.

To compare the ability of hydrophobic scales and the MPr score to predict whether residues are in EM or TM regions, we extended the definition of MPr score in Eq. (8) of the main text to hydrophobicity and defined the hydrophobicity score $\mathcal{H}_i$ of residue i as the weighted average of the hydrophobicity value over 5-residue sequence windows $[i-2, i+2]$:

$$\mathcal{H}_i = \frac{1}{1+2\gamma+2\beta}\left(\gamma\ {\rm Hyd}_{i-2} + \beta\ {\rm Hyd}_{i-1} + {\rm Hyd}_i + \beta\ {\rm Hyd}_{i+1} + \gamma {\rm Hyd}_{i+2}\right) \tag{1}$$

${\rm Hyd}_i$ is the hydrophobicity of residue i according to a given hydrophobicity scale. Similarly to what we did for the MPr score which is described in sections 2.3-2.5 of the main text, we chose the weighting parameters β and γ to minimize the level of weaknesses in the the $\mathcal{D}_{\rm mem}$ data set, and optimized a threshold value to compute the BACC score of the EM/TM localization prediction in terms of $\mathcal{H}_i$.

The BACC scores of the different hydrophobicity scores and the MPr score are reported in Table S3. We clearly see that the MPr score, with a BACC score of almost 0.9, is much better than the hydrophobicity scores for predicting EM/TM localization. Moreover, we plotted in Fig. S2 the distributions of the per-residue hydrophobicity scores and of the MPr score, separately for EM and TM regions. The two distributions are clearly separated when computed with the MPr score, in contrast to what happens with the hydrophobicity scores. This further confirms the superiority of the MPr score for EM/TM classification.

	Reference	BACC
$\mathcal{H}$ score	[7]	0.71
$\mathcal{H}$ score	[8]	0.68
$\mathcal{H}$ score	[9]	0.72
$\mathcal{H}$ score	[10]	0.72
MPr score	here	0.88

Table S3. BACC score of EM/TM classification using the per-residue MPr score and the hydrophobicity score $\mathcal{H}$ defined in Eq. (1) using the hydrophobicity scales in the four cited articles. The BACC score is computed for all residues i in the $\mathcal{D}_{\rm mem}$ data set.

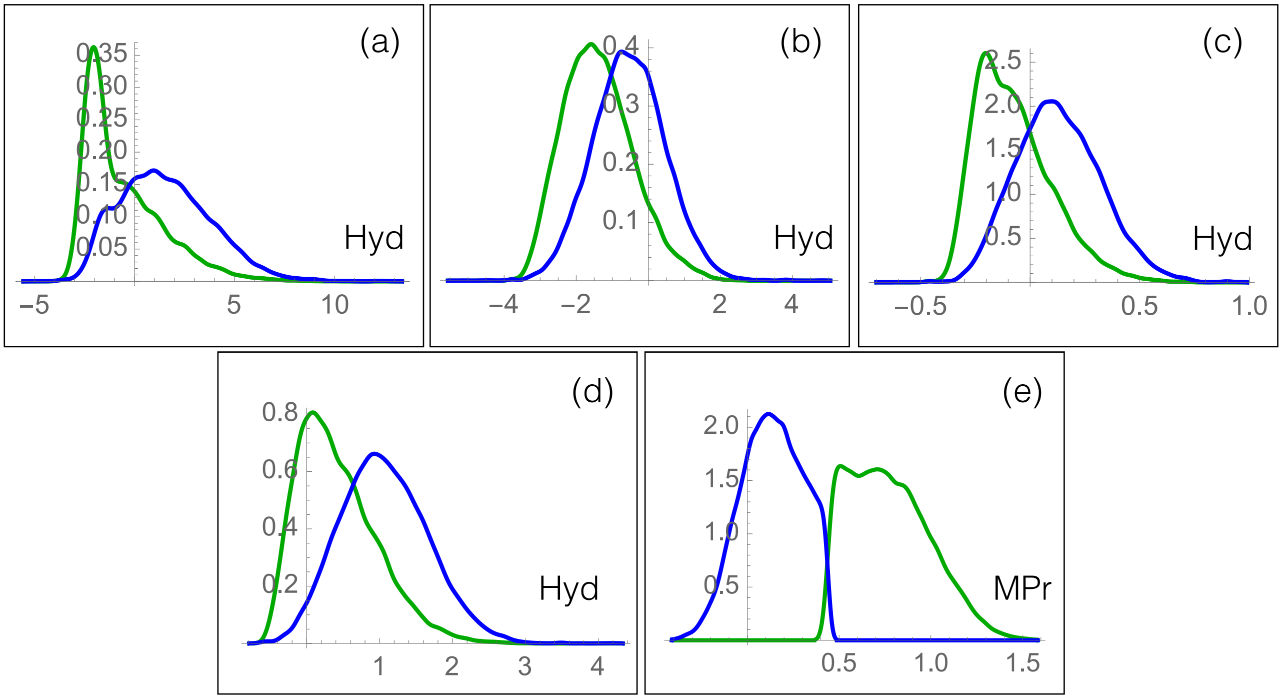

Figure S2. Distribution of per-residue $\mathcal{H}$ hydrophobicity scores (a)-(d) and MPr scores (e) for the residues from the $\mathcal{D}_{\rm mem}$ data set which belong to TM regions (in green) and to EM regions (in blue). The hydrophobicity scales are taken from the references: (a) [7], (b) [8], (c) [9], and (d) [10] (d).

S3.2 Relation between MPr score and bilayer Z-depth

Here we analyzed the relation between the MPr score and the Z-depth of each residue, defined as the distance between the residue side chain centroid and the plane parallel to the membranes cutting the bilayer into two equal parts. The result is shown in Fig. S3.

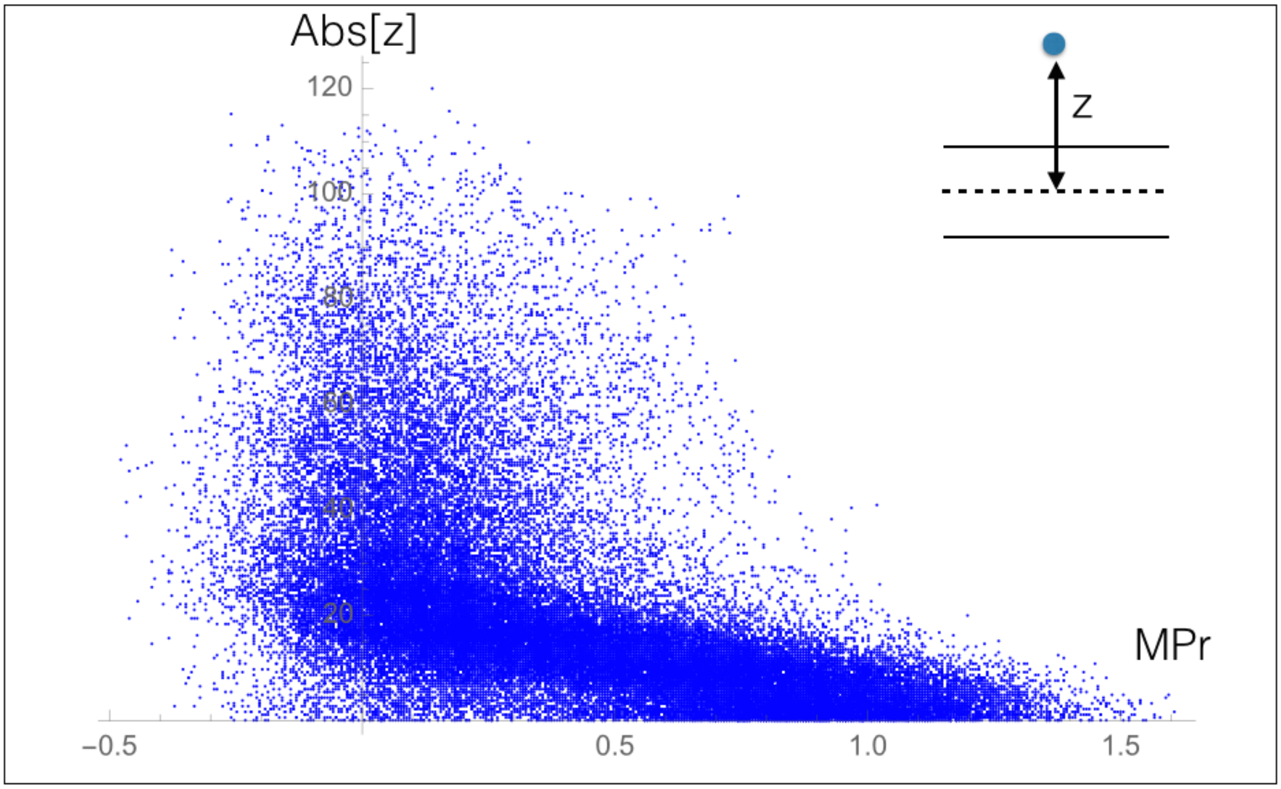

Figure S3. Absolute value of the lipid bilayer depth Z as a function of the per-residue MPr score for all residues belonging to the $\mathcal{D}_{\mathrm{mem}}$ data set. The Z-depth is schematically depicted in the upper right corner.

S3.3 Relation between MPr scores and normalized B-factors

In X-ray crystallography, B-factors (also known as temperature factors) reflect the attenuation of X-ray scattering caused by thermal motions and primarily serve as a measure of precision of mean atomic positions derived from the crystal data [11]. They are related to atomic flexibility, but only imperfectly [12, 13, 14, 15]. It is important to recall that crystallized proteins have drastically compromised atomic movements and that the B-factors are only reduced representations of the true cellular dynamics of the biomolecules. The fact that lipid bilayers are 'fluid mosaic' in nature [16] further adds to the complexity. It is also known that B-factors may not be compared between different protein structures. To be able to reliably compare the B-factors of different structures, they need to be properly normalized, as discussed in section 2.7 of the main manuscript. We considered two types of normalization schemes, the zero-mean-unit-variance scheme and the min-max-scaling scheme.

We computed the Pearson correlation coefficients r between the MPr scores and the normalized per-residue B-factors separately for the data sets $\mathcal{D}_{\text{mem}}$, $\mathcal{D}_{\text{mem}}^{\text{TM}}$, $\mathcal{D}_{\text{mem}}^{\text{EM}}$ and $\mathcal{D}_{\text{glob}}$. We also split $\mathcal{D}_{\text{glob}}$ into the subsets $\mathcal{D}_{\text{glob}}^{\text{core}}$ and $\mathcal{D}_{\text{glob}}^{\text{surface}}$, with the former containing the core residues with solvent accessibility$<$20% and the latter, surface residues with solvent accessibility$\geq$20%. We also considered separately the main-chain and side-chain B-factors.

The results are shown in Table S4. The best correlation coefficients are equal to -0.28 and are thus rather low. In membrane proteins, MPr scores and B-factors were found to be better anticorreled for TM residues than for EM residues. In globular proteins, the anticorrelation is better for core residues than for surface residues. The correlations of main-chain B-factors were all slightly better than the correlations of side-chain B-factors. Almost no difference was observed between the two B-factor normalization schemes. All correlation values were found statistically significant at the 99% level (p-value $< 10^{-5}$).

Data set	B-factor type	$r_{mean-var}$	$r_{min-max}$	N_{res}
$\mathcal{D}_{\text{mem}}$	main chain	-0.27	-0.28	51,141
$\mathcal{D}_{\text{mem}}^{\text{TM}}$	main chain	-0.20	-0.20	20,680
$\mathcal{D}_{\text{mem}}^{\text{EM}}$	main chain	-0.13	-0.16	30,461
$\mathcal{D}_{\text{mem}}$	side chain	-0.25	-0.26	51,233
$\mathcal{D}_{\text{mem}}^{\text{TM}}$	side chain	-0.19	-0.20	20,708
$\mathcal{D}_{\text{mem}}^{\text{EM}}$	side chain	-0.11	-0.13	30,525
$\mathcal{D}_{\text{glob}}$	main chain	-0.25	-0.25	1,159,619
$\mathcal{D}_{\text{glob}}^{\text{core}}$	main chain	-0.28	-0.28	454,971
$\mathcal{D}_{\text{glob}}^{\text{surface}}$	main chain	-0.16	-0.16	704,648
$\mathcal{D}_{\text{glob}}$	side chain	-0.24	-0.25	1,161,517
$\mathcal{D}_{\text{glob}}^{\text{core}}$	side chain	-0.24	-0.24	455,296
$\mathcal{D}_{\text{glob}}^{\text{surface}}$	side chain	-0.13	-0.13	706,221

Table S4. Pearson correlation coefficient r between MPr scores and normalized B-factors of all residues in the subsets of membrane and globular proteins indicated in the first column; N_{res} denotes the number of residues pertaining to each subset. The r values are given for both side-chain and main-chain B-factors, and for the two normalization schemes described in section 2.7 of the main manuscript, the zero-mean-unit-variance scheme ($r_{mean-var}$) and the min-max-scaling ($r_{min-max}$) scheme.

S4. EM/TM residue classification

As described in sections 2.6 and 3.4 of the main text, we used the MPr score to predict whether a given residue is in an EM or TM region. Here we show the ROC curve of this binary classification (Fig. S4), which represents the specificity as a function of the sensitivity:

$$\text{Sensitivity} = \frac{TP}{TP + FN} \tag{2}$$

$$\text{Specificity} = \frac{TN}{TN + FP} \tag{3}$$

The true positives (TP) and the true negatives (TN) are the correctly predicted TM and EM residues, respectively, and the false positives (FP) and false negatives (FN), the wrongly predicted TM and EM residues, respectively. The Area Under the Curve (AUC) is equal to 0.94.

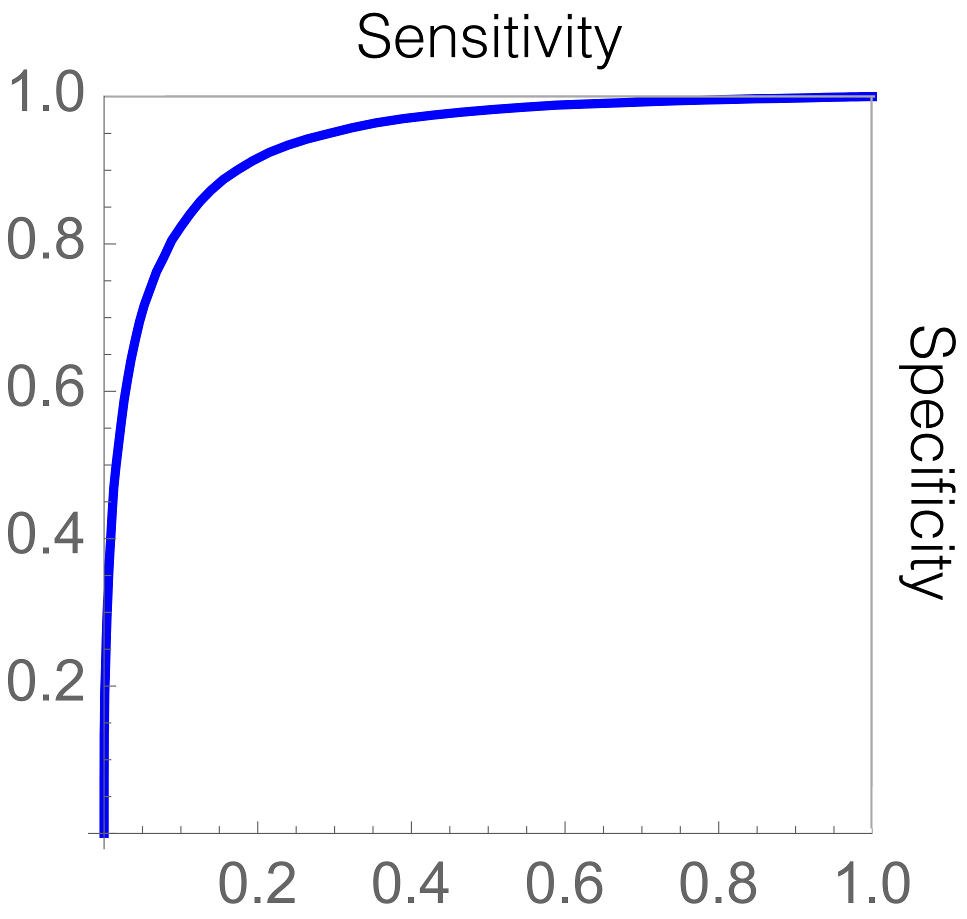

Figure S4. ROC curve of the EM/TM residue classification.

S5. Embedding in the lipid membrane: BRANEart versus OPM

We show in Fig. S5 four examples of classifications of EM and TM residues by OPM [17] and BRANEart.

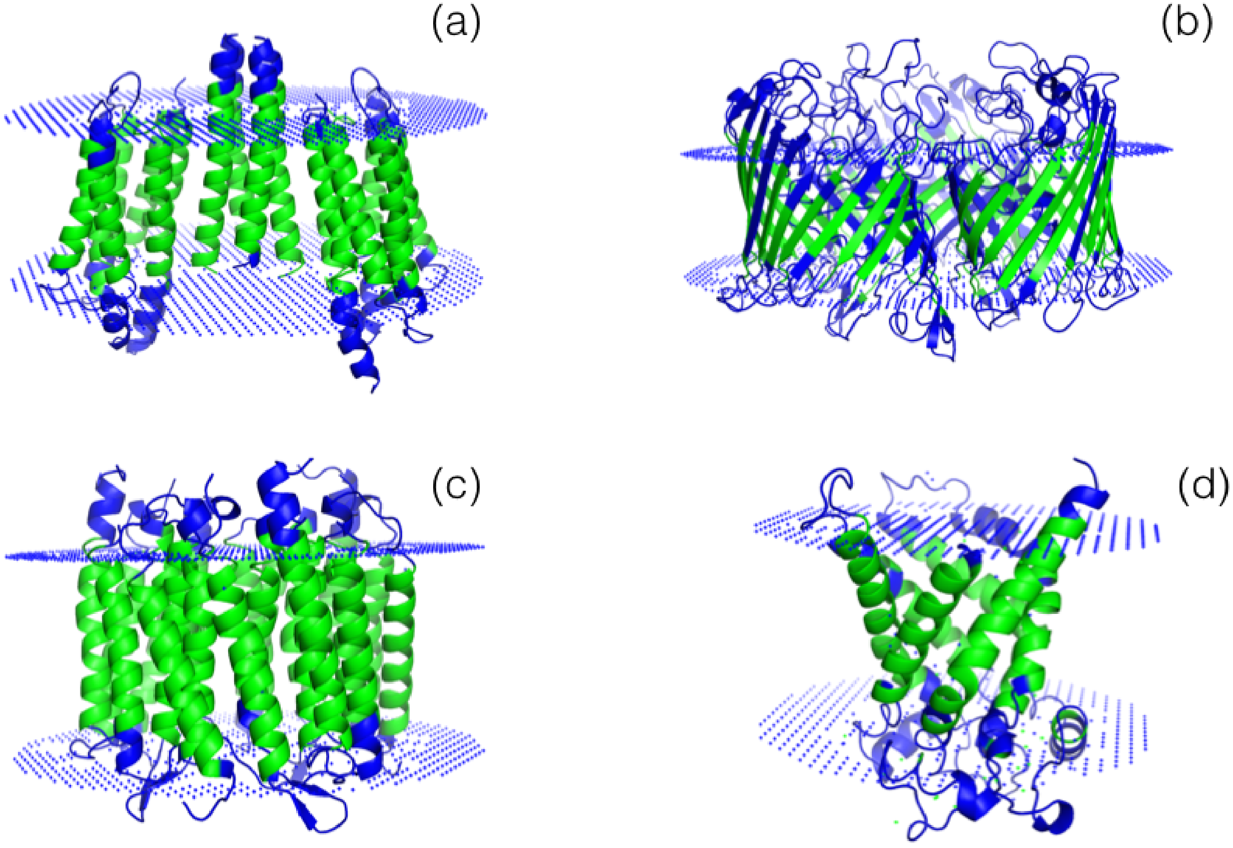

Figure S5. Comparison between BRANEart and OPM classifications of EM/TM residues. The planes represent the limits of the lipid bilayer predicted by OPM; all residues between the two planes are predicted as TM and all residues outside the planes as EM. BRANEart classification is indicated by colors, with blue and green residues identifying EM and TM residues, respectively. The proteins shown have the PDB codes: (a) 1H2S, (b) 1A0S, (c) 1M0L and (d) 1OKC.

References

[1] Marianne J Rooman, Jean-Pierre A Kocher, and Shoshana J Wodak. Prediction of protein backbone conformation based on seven structure assignments: influence of local interactions. *Journal of Molecular Biology*, 221(3):961–979, 1991.

[2] Jean-Pierre A Kocher, Marianne J Rooman, and Shoshana J Wodak. Factors influencing the ability of knowledge-based potentials to identify native sequence-structure matches. *Journal of Molecular Biology*, 235(5):1598–1613, 1994.

[3] Qingzhen Hou, Fabrizio Pucci, François Ancien, Jean Marc Kwasigroch, Raphaël Bourgeas, and Marianne Rooman. SWOTein: A structure-based approach to predict stability Strengths and Weaknesses of prOTEINs. *Bioinformatics*, btab034(Epub ahead of print), 2021.

[4] Yves Dehouck, Dimitri Gilis, and Marianne Rooman. A new generation of statistical potentials for proteins. *Biophysical journal*, 90(11):4010–4017, 2006.

[5] Mame Ndew Mbaye, Qingzhen Hou, Sankar Basu, Fabian Teheux, Fabrizio Pucci, and Marianne Rooman. A comprehensive computational study of amino acid interactions in membrane proteins. *Scientific reports*, 9(1):1–14, 2019.

[6] Yves Dehouck, Aline Grosfils, Benjamin Folch, Dimitri Gilis, Philippe Bogaerts, and Marianne Rooman. Fast and accurate predictions of protein stability changes upon mutations using statistical potentials and neural networks: PoPMuSiC-2.0. *Bioinformatics*, 25(19):2537–2543, 2009.

[7] DM Engelman, TA Steitz, and A Goldman. Identifying nonpolar transbilayer helices in amino acid sequences of membrane proteins. *Annual review of biophysics and biophysical chemistry*, 15(1):321–353, 1986.

[8] C Preston Moon and Karen G Fleming. Side-chain hydrophobicity scale derived from transmembrane protein folding into lipid bilayers. *Proceedings of the National Academy of Sciences*, 108(25):10174–10177, 2011.

[9] Julia Koehler, Nils Woetzel, René Staritzbichler, Charles R Sanders, and Jens Meiler. A unified hydrophobicity scale for multispan membrane proteins. *Proteins: Structure, Function, and Bioinformatics*, 76(1):13–29, 2009.

[10] Tara Hessa, Hyun Kim, Karl Bihlmaier, Carolina Lundin, Jorrit Boekel, Helena Andersson, IngMarie Nilsson, Stephen H White, and Gunnar von Heijne. Recognition of transmembrane helices by the endoplasmic reticulum translocon. *Nature*, 433(7024):377–381, 2005.

[11] Steve P Meisburger, David A Case, and Nozomi Ando. Diffuse X-ray scattering from correlated motions in a protein crystal. *Nature communications*, 11(1):1–13, 2020.

[12] T Ackermann, CL Brooks III, M Karplus, and BM Pettitt. Proteins: a theoretical perspective of dynamics, structure and thermodynamics, volume lxxi. *advances in chemical physics, John Wiley & Sons, New York*, pages 96–96, 1988.

[13] Krzysztof Kuczera, John Kuriyan, and Martin Karplus. Temperature dependence of the structure and dynamics of myoglobin: a simulation approach. *Journal of molecular biology*, 213(2):351–373, 1990.

[14] Saul R Trevino, Stephanie Schaefer, J Martin Scholtz, and C Nick Pace. Increasing protein conformational stability by optimizing β-turn sequence. *Journal of molecular biology*, 373(1):211–218, 2007.

[15] Zhoutong Sun, Qian Liu, Ge Qu, Yan Feng, and Manfred T Reetz. Utility of B-factors in protein science: interpreting rigidity, flexibility, and internal motion and engineering thermostability. *Chemical reviews*, 119(3):1626–1665, 2019.

[16] S Jonathan Singer and Garth L Nicolson. The fluid mosaic model of the structure of cell membranes. *Science*, 175(4023):720–731, 1972.

[17] Mikhail A Lomize, Irina D Pogozheva, Hyeon Joo, Henry I Mosberg, and Andrei L Lomize. OPM database and PPM web server: resources for positioning of proteins in membranes. *Nucleic acids research*, 40(D1):D370–D376, 2012.
